# Case Report: *Bordetella bronchiseptica* Meningoencephalomyelitis in a Dog

**DOI:** 10.3389/fvets.2022.852982

**Published:** 2022-04-05

**Authors:** Helena Rylander, Dylan M. Djani, Starr Cameron

**Affiliations:** University of Wisconsin-Madison, Madison, WI, United States

**Keywords:** bacterial meningitis and meningoencephalitis, neurology, cerebrospinal fluid (CSF), *Bordetella bronchiseptica*, brain, spinal cord

## Abstract

A 15-month-old male neutered Wirehaired Pointer mixed-breed dog presented with fever and cervical pain. Cerebrospinal fluid (CSF) analysis showed neutrophilic pleocytosis with intracellular bacteria, and culture of CSF grew *Bordetella bronchiseptica*. The patient became non-ambulatory 3 days after CSF collection. He was treated with low-dose prednisone for 3.5 months and doxycycline for 1 year. Recheck CSF analysis 1 month after diagnosis showed reduction of inflammation and 3 months after diagnosis revealed only increased protein. The patient improved neurologically over several months and was weakly ambulatory 5 months and fully ambulatory 7 months after diagnosis. Whole genome sequencing of the bacterial isolate and a live modified intranasal vaccine similar to the one the dog had been vaccinated with 7 weeks before diagnosis was similar but not an exact match. Bacterial meningitis should be considered, and culture of CSF is recommended, in cases of neutrophilic pleocytosis of CSF.

## Introduction

Bacterial meningoencephalomyelitis (BMEM) has rarely been reported in veterinary medicine (1). The most common routes of infection are hematogenous spread from a distant focus, direct invasion (trauma and bite wound), or contiguous spread such as from an otitis interna or nasal infection. A few case reports describe BMEM as primary infections or opportunistic infections secondary to other diseases (2–8). In addition, review articles describe BMEM and list various infectious organisms (9–11). Our report is the first of a BMEM caused by *Bordetella bronchiseptica* in a dog.

## Case Description

A 15-month-old male neutered Wirehaired Pointer mixed-breed dog weighing 16.4 kg presented to the University of Wisconsin Veterinary Care (UWVC) for a 3.5-week history of cervical pain. The dog had been treated with carprofen [2 mg/kg Per Os (PO) q12h] for acute cervical pain and a fever of 105°F 3.5 weeks before presentation. Two weeks before presentation, the carprofen was discontinued, and he was started on prednisone (0.6 mg/kg PO q12h for 5 days and then 0.6 mg/kg PO q24h), gabapentin (12 mg/kg PO q8h), and tramadol (0.3 mg/kg PO q8h). Diarrhea developed after a few days. Five days before presentation, gabapentin and tramadol were discontinued, and acetaminophen (1.8 mg/kg PO q8h), ondansetron (0.5 mg/kg PO q8h), omeprazole (1.2 mg/kg PO q24h), and pregabalin (3 mg/kg PO q12h) were started. He had been off prednisone for 2 days at the time of the examination. On the day before initial neurologic examination, the dog was admitted to UWVC for worsening cervical pain and was placed on fentanyl [2–5μg/kg/h intravenously (IV)] overnight. In addition, he had been treated for gastroenteritis and a honking cough 5 months before presentation.

On physical examination, the dog had a body condition score of 5/9. All findings were within normal limits except for cervical pain and reluctance moving the neck. Neurologic examination was within normal limits except for absent paw placement in both pelvic limbs, a slow stiff gait, and cervical pain on palpation and manipulation of the neck. The paw placement deficits were attributed to the fentanyl treatment. On the basis of the signalment, history, and presentation, the top differential diagnosis was steroid responsive meningitis arteritis, although a meningoencephalomyelitis of unknown origin or an infectious cause could not be ruled out.

A chemistry panel was performed and was unremarkable. Complete blood count showed a leukocytosis with a mature neutrophilia (white blood cell count of 18,500 cells/μl, reference range of 5,000–14,000; segmented neutrophils of 1,170 cells/μl, reference range of 2,600–10,000; monocytes of 1,300 cells/μl, reference range of 100–900; and basophils of 200 cells/μl, reference range of 0–100).

The dog was routinely anesthetized for cerebrospinal fluid (CSF) collection. The CSF from the cerebellomedullary cistern was milky white and cloudy in appearance and showed an increase in total protein content (320 mg/dl; reference range of <25), mild blood contamination (red blood cell count of 320/μl, reference of 0) and a neutrophilic pleocytosis (total nucleated cell count of 10,750/μl, reference range <5; 83% neutrophils, reference range of 0–10; 9% lymphocytes, reference range of 50–60; and 8% mononuclear cells, reference range of 40–50). The lumbar CSF was blood tinged and hazy and had an increase in protein content (656 mg/dl; reference range of <35), mild blood contamination (red blood cell count of 1,215/μl), and a neutrophilic pleocytosis (total nucleated cell count of 140/μl, reference range of <5; 71% neutrophils, reference range of 0–10; 26% lymphocytes, reference range of 50–60; and 3% mononuclear cells, reference range of 40–50) (12). The CSF was cultured and prednisone (0.6 mg/kg PO q12h) and azathioprine (1.5 mg/kg PO q24h) were started for presumed steroid responsive meningitis arteritis (SRMA).

The following day, the dog appeared more ataxic. He was mildly obtunded and ambulatory with a mild paraparesis and moderate proprioceptive ataxia. Paw placement test was absent in the pelvic limbs and normal in the thoracic limbs. The change was initially attributed to the treatment with fentanyl constant rate infusion (CRI) overnight and the anesthesia.

**Figure 1:**
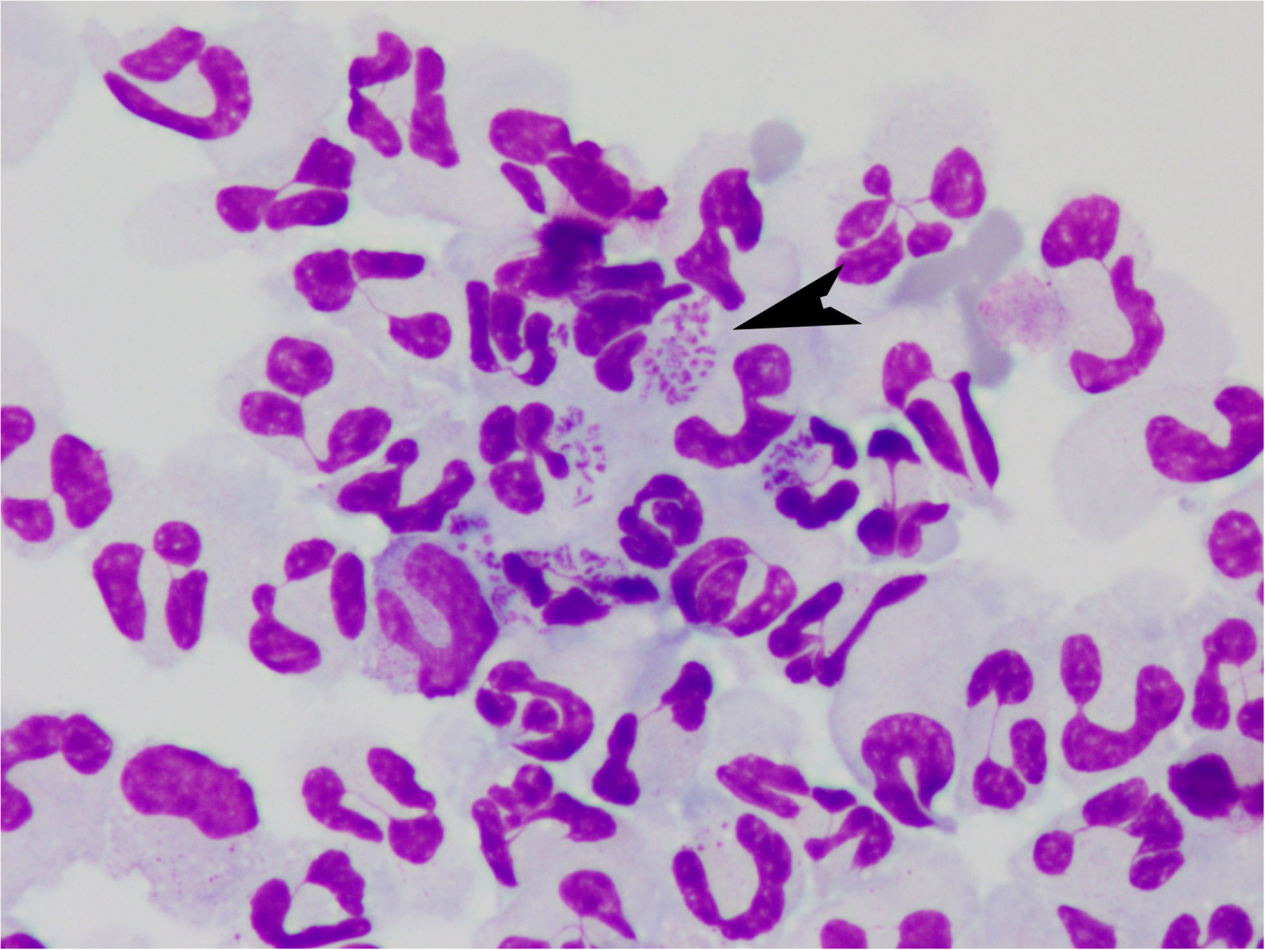
Cerebrospinal fluid from a dog with *Bordetella bronchiseptica* meningoencephalomyelitis. Note the intracellular bacteria (arrow). Modified Wright-Giemsa 1000X.

On further review of the CSF slides, there were a few intracellular basophilic short bacterial rods in the neutrophils (Figure 1). Culture of the CSF showed a moderate growth of *Bordetella bronchiseptica*. On further questioning the owner, the dog had received a modified live Bordetella vaccine intranasally 3.5 weeks before onset of clinical signs. The azathioprine was discontinued.

The dog was discharged the following day with prednisone (0.45 mg/kg PO q24h), doxycycline (4.5 mg/kg PO q12h), Proviable^TM^ probiotic capsule (1 capsule PO q24), and continued ondansetron and acetaminophen at previous doses. At the time of discharge, he was mildly obtunded and ambulatory paraparetic with absent paw placement in both pelvic limbs. He became non-ambulatory paraparetic 3 days after the spinal fluid collection.

One month after diagnosis, the dog was able to stand on his own but remained non-ambulatory. He was fecally incontinent and able to urinate on his own, but manual expression of the urinary bladder by the owner was required for complete emptying. The patient had regular visits to a physical therapist for rehabilitation and had a home exercise program. On neurologic examination he was bright, alert, and responsive (BAR), with normal cranial nerve examination. He was non-ambulatory paraparetic with good voluntary motor function. Paw placement was absent in both pelvic limbs and normal in the thoracic limbs. Spinal reflexes were normal, except for crossed extensor reflex in the pelvic limbs. There was no pain noted on palpation of the spine including the neck, and there was full range of motion of the neck.

Repeat CSF analysis was done to evaluate response to treatment. The CSF (cisternal collection) showed an increased total protein (53 mg/dl), a red blood cell count of 2/μl, and a lymphocytic pleocytosis (total nucleated cell count of 62/μl, 7% neutrophils, 55% lymphocytes, and 38% mononuclear cells). Lumbar collection of CSF showed an increased total protein (324 mg/dl), a red blood cell count of 3/μl, and a lymphocytic pleocytosis (total nucleated cell count of 16/ μl, 70% lymphocytes, and 30% mononuclear cells). No organisms were seen. The interpretation was chronic inflammation or ongoing antigen stimulation.

The dose of prednisone was increased (0.45 mg/kg PO q12h), and the doxycycline and Proviable^TM^ were continued at the previous doses.

**Figure 2:**
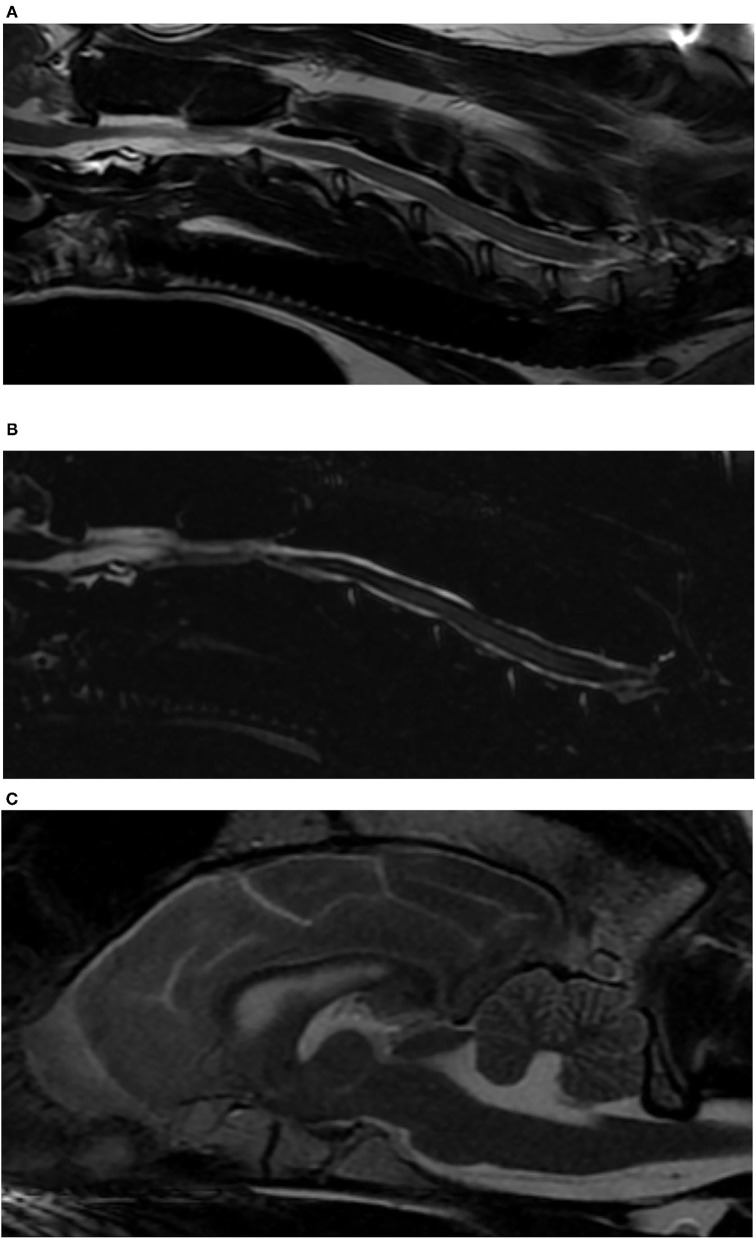
Magnetic resonance images of a dog with *Bordetella bronchiseptica* meningoencephalomyelitis. **(A)** Sagittal T2-weighted image of the cervical spinal cord. Note the intramedullary hyperintensity at the level of C4–C6 vertebral bodies and the mildly enlarged central canal at the level of C6 vertebral body. **(B)** Sagittal MYELO haste image of the cervical spinal cord. Note the thinning of the dorsal subarachnoid CSF column at the level of C5 vertebral body, and the previously described intramedullary hyperintensity. **(C)** Sagittal T2-weighted image of the brain. Note the enlarged ventricular system.

Three months after diagnosis, the dog was BAR, a ventral strabismus oculus dexter (OD) was present, and he was non-ambulatory tetraparetic with the pelvic limbs appearing weaker than the thoracic limbs. Paw placement was absent in both pelvic limbs and the right thoracic limb. The patella reflexes were increased and there was a crossed extensor reflex bilaterally in the pelvic limbs. There was no pain on vertebral column palpation. Gamma-glutamyl transferase was mildly elevated (55 U/L; reference range of 5–16), and alkaline phosphatase was elevated (959 U/L; reference range of 20–157). White blood cell count was within normal limits (WNL), and there was a mild non-regenerative anemia (hematocrit 33%; reference range of 39–57). Given the clinical progression and change in neurologic status, a magnetic resonance imaging (MRI) to evaluate for structural lesions of the cervical and thoracic spine and brain were performed (GE Healthcare, 1.5 Tesla, Milwaukee, Wisconsin) (Figure 2). The images obtained of the thoracolumbar spine were T2-weighted (T2W) sagittal, T1-weighted (T1W)-pre and -post contrast sagittal, and MYELO-haste. Images obtained from the brain were T2W sagittal and transverse, T1W-post contrast sagittal, transverse and dorsal, fluid attenuation inversed recovery (FLAIR) sagittal, and images obtained from the cervical spine were T2W sagittal and transverse, T1W-post contrast sagittal and transverse, and MYELO haste sagittal. There was mild patchy intramedullary T2W hyperintensity from C3 to C7 spanning a larger portion of the spinal cord with amorphous margins from C4 to C6 and mild central canal dilation. There was mild thinning of the CSF at this level, best seen on myelo-HASTE. In the brain, there was mild periventricular FLAIR hyperintensity tracking along the right lateral ventricle, and the lateral and fourth ventricles were mildly dilated.

The lumbar CSF performed at this time contained an elevated protein with mild blood contamination and a normal cell count (protein of 367 mg/dl, red blood cell count of 42/μl, total nucleated cell count of 5/μl, 4% neutrophils, 82% lymphocytes, and 14% mononuclear cells). Cisternal CSF showed a mildly elevated protein with a normal cell count (total protein of 49 mg/dl, red blood cell count of 1/μl, and total nucleated cell count of 2/μl). Culture of CSF showed no growth.

The dose of prednisone was reduced (0.3 mg/kg PO q12h for 5 days, then 0.3 mg/kg PO q24h for 7 days, then discontinued). The doxycycline and Proviable^TM^ were continued.

Over the next 4 weeks, the dog continued to improve in attitude—being more playful and alert at home. On recheck 4 weeks later (5 months after diagnosis), he was able to take 10 steps with a tetraparesis worse in the pelvic limbs and generalized proprioceptive ataxia. The remainder of the examination was unchanged.

Seven months after diagnosis, the dog was ambulatory with a spastic paraparesis and a generalized proprioceptive ataxia. The fecal incontinence and inability to fully void resolved 11 months after diagnosis, and he was able to walk for longer distances—still with a paraparesis and generalized proprioceptive ataxia. The crossed extensor reflex in the pelvic limbs was still present. One year after diagnosis, the dog was ambulatory with a spastic paraparesis and a mild to moderate proprioceptive ataxia in the pelvic limbs. The doxycycline was discontinued.

Whole genome sequencing of a Bordetella vaccine similar to what the dog received in March of 2020 and the *Bordetella bronchiseptica* cultured from the CSF showed that the isolates were 50,525 single nucleotid polymorphism (SNP) apart, suggesting that the isolates were very close but not completely identical.

## Discussion

Bacterial meningitis is relatively rare in dogs. To the authors' knowledge, this is the first report of *Bordetella bronchiseptica* meningoencephalomyelitis in a dog. *Bordetella bronchiseptica* is one of the causative agents of canine infectious tracheobronchitis and is a gram-negative, aerobic coccobacillus. Transmission occurs after direct contact with infected dogs or contaminated material from aerosolized microdroplets. After the 6 days of incubation period, the bacteria attach to and replicate on the cilia of respiratory epithelium. Toxin produced by the bacteria impair phagocytic function and induce ciliostasis. The bacteria can invade host cells and thus avoid immunologic defense mechanisms and establish a persistent infection. The bacteria persist in the epithelium for 3 months before it is cleared by local antibody production. The organism has been isolated from the upper respiratory tracts from clinically healthy dogs. Dogs recently vaccinated may still develop clinical signs. Adverse reactions reported after vaccination include a cough or nasal discharge 2–5 days after inoculation (13).

Steroid responsive meningitis arteritis has been shown to be more prevalent in some breeds, among them the Wirehaired Pointing Griffon (14). The initial presentation with fever, cervical pain, and leukocytosis and a normal neurologic examination made the diagnosis of SRMA the most likely in our patient. After reviewing the CSF slides, intracellular bacteria were seen in the neutrophils. Culture of the CSF showed growth of *Bordetella bronchiseptica*. Although positive cultures on CSF has been reported in only 17–30% of confirmed bacterial meningitis, culture of CSF is recommended in cases of neutrophilic pleocytosis in the CSF (10).

Our patient received an intranasal modified live Bordetella vaccine 3.5 weeks before onset of cervical pain, which was 7 weeks before the diagnosis of *Bordetella Bronchiseptica* BMEM. Passage from the nasal cavity to the brain is possible even with large molecules, such as vaccines (>900 Daltons), via the olfactory pathways or trigeminal pathways, after overcoming the nasal mucosal barrier (15). The genome sequencing of the isolated bacteria and the bacteria from the vaccine showed that they were molecularly very close but not the exact same strain. There are a few possible explanations for this finding. First, the bacteria from the vaccine may have mutated within the patient before CSF collection, making the isolate slightly different. Second, the vaccine tested was the same manufacturer but from a different year and different batch than what our patient received. Therefore, it is possible that, if the vaccine that was actually administered was tested, then the isolates may have been more or less similar. Last, the infection and the vaccine may not have been the same strain, meaning that the vaccine did not cause the infection.

The choice of antibiotic was based on susceptibility of the isolate and that doxycycline has good penetration into the central nervous system. Treatment with prednisone is controversial in infectious disease but has been recommended at low doses for the short-term in infectious meningoencephalomyelitis to reduce the inflammatory response as the organism dies from the treatment (16). The risk with longtime treatment is that steroids reduce the permeability of the blood brain barrier and thus decrease the penetration of antibiotics. Treatment with antibiotics was 1 year in our patient to reduce the risk of recurrence, although there are no guidelines for how long to treat dogs with BMEM. A negative culture of the CSF 3 months after diagnosis was promising. However, a residual bacterial infection could not fully be ruled out given the low success of culture of CSF, as well as our patient still being very clinically affected. As the inflammation resolves, the blood brain barrier function improves and reduces transport of medication to the brain.

The worsening of neurologic signs and the findings on MRI may have been due to damage to the spinal cord parenchyma from the infection, although as stated above, a residual bacterial infection could not fully be ruled out.

The more common *Bordetella* species that infects humans is *Bordetella pertussis*, which is the causative agent for whooping cough. *Bordetella bronchiseptica* is infrequently reported as cause for airway infection in immunocompromised people, and only four reports of meningitis have been reported in people. It was diagnosed in one patient secondary to a traumatic CSF leak after a fall, in one patient who underwent surgery after having been kicked by a horse, and in two patients after brain surgery. All patients had household pets or were in close contact with dogs (17–20).

The mortality rate in people with bacterial meningitis is 5–40%, and 30% of survivors have permanent neurologic deficits (1). A review article in dogs concluded that 10% of dogs have residual signs and 6% of dogs relapse with BMEM (10). Our patient continued to improve over the first year but had permanent neurologic deficits.

In conclusion, we report the first case of BMEM caused by *Bordetella bronchiseptica* in a dog. BMEM should be ruled out in cases presenting with a fever and neutrophilic pleocytosis on CSF. Culture of CSF in case of neutrophilic pleocytosis is highly recommended, even if the yield for positive culture is low. Recovery from BMEM may be slow with residual neurologic deficits.

## Data Availability Statement

The SNP was done at a third party, CosmosID laboratory. Questions can be directed to info@cosmosid.com.

## Ethics Statement

Written informed consent was obtained from the owners of the dog for the publication of this case report.

## Author Contributions

HR, DD, and SC all handled the case in the clinic and helped writing the manuscript. All authors contributed to the article and approved the submitted version.

## Conflict of Interest

The authors declare that the research was conducted in the absence of any commercial or financial relationships that could be construed as a potential conflict of interest.

## Publisher's Note

All claims expressed in this article are solely those of the authors and do not necessarily represent those of their affiliated organizations, or those of the publisher, the editors and the reviewers. Any product that may be evaluated in this article, or claim that may be made by its manufacturer, is not guaranteed or endorsed by the publisher.

## Abbreviations

BMEM: bacterial meningoencephalomyelitis
CSF: cerebrospinal fluid
FLAIR: fluid attenuation inversed recovery
T1W: T1-weighted
T2W: T2-weighted.
